# Resveratrol exerts an anti-apoptotic effect on human bronchial epithelial cells undergoing cigarette smoke exposure

**DOI:** 10.3892/mmr.2014.2925

**Published:** 2014-11-11

**Authors:** LI ZHANG, XIALING GUO, WANG XIE, YUPING LI, MIAO MA, TING YUAN, BAILING LUO

**Affiliations:** 1 Department of Respiratory Medicine, Xiangya Hospital, Central South University, Changsha, Hunan 410008, P.R. China; 2 Department of Critical Emergency Medicine, The Second Xiangya Hospital, Central South University, Changsha, Hunan 410007, P.R. China

**Keywords:** chronic obstructive pulmonary disease, SIRT1, ORP150, apoptosis, resveratrol, endoplasmic reticulum stress

## Abstract

Cigarette smoke can cause endoplasmic reticulum stress and induce apoptosis, both of which are important pathogenic factors contributing to chronic obstructive pulmonary disease. The aim of the present study was to produce a cigarette smoke extract (CSE)-induced apoptosis human bronchial epithelial cell (HBEpC) model, to investigate the protective effects of resveratrol (RES). The role of oxygen-regulated protein 150 (ORP150) in the RES-induced activation of Sirtuin 1 (SIRT1) was additionally studied. Cultured HBEpCs were initially treated with CSE to induce apoptosis, followed by an incubation either with or without RES. Numerous techniques were used to evaluate the outcomes of the present study, including cell counting kit-8 assay, quantitative polymerase chain reaction, western blotting, Hoechst 33342 staining and AnnexinV-PI flow cytometry apoptosis analyses, and gene knockdown. It was identified that 24 h 2% CSE incubation induced apoptosis in HBEpC, accompanied by an overexpression of the apoptosis molecular markers CCAAT-enhancer-binding protein homologous protein, caspase 4 and caspase 3. Pre-treatment of the cells with RES markedly alleviated the severity of apoptosis, as confirmed by apoptosis analyses and the expression levels of the apoptosis molecular markers. SIRT1 was shown to be overexpressed following RES treatment. However, following the gene knockdown of ORP150, the anti-apoptotic effects of RES were significantly attenuated. The results of the present study demonstrate that RES may have a protective effect against CSE-induced apoptosis, and a molecular pathway involving SIRT1 and ORP150 may be associated with the anti-apoptotic functions of RES in HBEpC.

## Introduction

Cigarette smoke contains numerous harmful compounds, including nicotine, heavy metals, free radicals, oxidants, and reactive oxygen and nitrogen species. Smoking is known to be the main risk factor for chronic obstructive pulmonary disease (COPD) (1). Apoptosis of the structural cells of the lung, is a pathogenetic event in the pathogenesis of COPD (2). In cases of severe and prolonged smoking, cigarette smoke is capable of inducing endoplasmic-reticulum (ER) stress, which causes the unfolded protein response and apoptosis in pulmonary cells (3–5).

Sirtuin 1 (SIRT1) is a well-known longevity gene, that regulates senescence, stress resistance, inflammation and DNA repair by deacetylation of intracellular signaling molecules and histones (6). Previous studies have shown that the therapeutic effects of SIRT1 can be achieved following gene activation by resveratrol (RES) (7,8). The vasoprotective effects of RES have mainly been observed against apoptosis induced by insults including hypoxia and cigarette smoke (CS). Evidence for the anti-apoptotic effects of RES has also come from retinal and hepatic cells undergoing different insults including ethanol, palmitate and a specific antibody (9–12). Furthermore, in the lungs of patients and murine models of COPD, SIRT1 has been shown to be downregulated (13,14), indicating the importance of intact, or upregulated SIRT1 function in the prevention of COPD.

Oxygen-regulated protein 150 (ORP150) is an important member of the 70kDa heat shock protein family. Knockdown of ORP150 has previously been shown to promote endoplasmic reticulum (ER)-stress, induce apoptosis and increase the expression of CCAAT-enhancer binding protein (CHOP), an ER-stress-induced apoptosis marker (15,16). Jung *et al* (17) previously reported that through the induction of ORP150, SIRT1 alleviated palmitate-induced ER-stress in HepG2 human hepatocytes. Furthermore, studies have shown that SIRT1 protects against CS-induced cellular senescence through the SIRT1-forkhead box O3 (FOXO3) axis (13,18). SIRT1 has also been demonstrated to be associated with the regulation of ORP150 gene expression, by FOXO1 (19).

Numerous studies have determined the anti-apoptotic effects of RES, in particular through its activation of SIRT1, which may be associated with ORP150. Therefore it was hypothesized in the present study that the anti-apoptotic effects of RES may be observed in human bronchial epithelial cells (HBEpC), and that ORP150 may be associated with the activation of SIRT1 by RES, resulting in an anti-apoptotic effect.

To evaluate the anti-apoptotic effects of RES on HBEpC, and to explore the role of ORP150 in the activation of SIRT1 by RES, the well-established cigarette smoke extract (CSE) apoptosis cell model in HBEpC was used. Techniques including cell culture, cell counting kit-8 assay, gene knockdown, quantitative polymerase chain reaction (qPCR), western blotting, and Hoechst 33342 staining and AnnexinV-PI flow cytometry apoptosis analyses were used throughout the study.

## Materials and methods

### CSE preparation and cell culture

CSE was prepared using a smoke machine as described by previous methods (20,21). The direct and side-stream smoke from a cigarette (Fu Rong Wang brand) was directed via a tube through 3 ml phosphate-buffered saline, using a peristaltic pump (Cole-Parmer, Vernon Hills, IL, USA). A spectrometer (Inifinite 200 PRO; Tecan, Männedorf, Switzerland) was used to determine the optical density of the extract at a wavelength of 450 nm. The 3 ml solution was determined as 100% CSE. In all of the experiments, freshly prepared CSE was used.

HBEpC were obtained from Xiangya Central Laboratory and were cultured in a humidified incubator containing 95% air and 5% CO_2_, at 37°C, in RPMI-1640 medium (Gibco-BRL, Carlsbad, CA, USA), supplemented with 10% heat-inactivated fetal bovine serum (FBS; Hyclone Laboratories, Inc., Logan, UT, USA). The cells were detached for subculture using 1% trypsin (Beyotime Institute of Biotechnology, Haimen, China). Once the cells had reached 80% confluence, they were seeded into six-well plates at a density of 1×10^5^ cells/well, and grown to 80% confluence, prior to being used for further experiments.

### Preparation of RES solution

RES (purity >99%), was purchased from Sigma-Aldrich (Shanghai, China). A stock solution of 105 μmol/l was made by dissolving 22.8 mg RES in 1 ml dimethyl sulfoxide. The final concentration of RES used in the present study was 20 μmol/l.

### Cell Counting kit-8 (CCK-8) assay

To determine a suitable concentration and duration of the CSE intervention, a CCK-8 assay was used to monitor cellular viability. The cultured HBEpC, at 80% confluence, were treated with CSE at various concentrations for 0, 6, 12, 18 and 24 h in 37°C in an incubator containing 95% air and 5% CO_2_. The CCK-8 assay reagent (Dojindo Laboratories, Kumamoto, Japan) was added to the culture media and incubated for 2 h. A micro-plate reader was used to measure the absorbance at 450 nm. Each assay was performed in triplicate.

### Analyses of apoptosis

To visualize the morphological changes of HBEpC during apoptosis, a Hoechst 33342 staining kit (Beyotime Institute of Biotechnology) was used, as described by previous methods (22). At the end of each experiment, the cells were stained with Hoechst 33342 (1 mg/ml) for 15 min. The cells were then observed under an IX71 inverted fluorescence microscope (Olympus, Tokyo, Japan).

To quantitatively analyze the rate of apoptosis of the cells, Annexin V-propidium iodide (PI) flow cytometry was performed using an Annexin V-fluorescein isothiocyanate (FITC) apoptosis detection kit (BD Biosciences, Franklin Lakes, NJ, USA). Following the treatment of the cells with RES, with or without CSE for 24 h, ~1×10^5^ cells were collected and resuspended in 500 μl Annexin V binding buffer (1X). Annexin V-FITC (5 μl) and PI (5 μl) were then added to each sample, followed by an incubation for 15 min at room temperature (25°C) in the dark. The cells were then quickly subjected to fluorescence-activated cell sorting analysis (FACSCalibur; BD Biosciences).

### qPCR

Total RNA was isolated from HBEpCs using TRIzol^®^ (Invitrogen Life Technologies, Beijing, China), according to the manufacturer’s instructions. cDNA was synthesized from the RNA using the Superscript II Reverse Transcription system (Invitrogen Life Technologies). qPCR was performed using a SYBR Green PCR kit (Takara Biotechnology Co. Ltd., Dalian, China) using an iCycler (ABI ViiATM7; Applied Biosystems, Carlsbad, CA, USA). The thermocycler parameters were set as follows: Step one, activation of the HotStartTaq DNA polymerase (Takara Biotechnology Co. Ltd.) at 95°C/30 sec; step two, PCR was performed for 40 cycles, denaturation at 95°C/5 sec, annealing at 60°C/34 sec; step three, fixed parameters set by the ABI ViiA 7 Fast Real-time PCR system (Applied Biosystems). GAPDH was used as an internal standard. The primer sequences were designed using the NCBI-Primer Basic Local Alignment Search Tool (National Institutes of Health, Bethesda, MD, USA) online tool and synthesized by Sangon Biotech, Shanghai, Co., Ltd. (Shanghai, China). The oligonucleotides used were as follows: SIRT1 forward, 5′-ATTCCAGCCATCTCTCTGTCAC-3′, and reverse, 5′-GTCTTGTATCTGTGCGACCTTG-3′; ORP150 forward, 5′-CAGAGGGAGAGAAGAAGCAGAA-3′, and reverse 5′-CAAGACCTGGACGGACTGAA-3′; GAPDH forward, 5′-AGAAGGCTGGGGCTCATTTG-3′, and reverse, 5′-AGGGGCCATCCACAGTCTTC-3′.

### Western blot analysis

Following cell culture, HBEpC were harvested and RIPA lysis buffer and 100 mM phenylmethanesulfonyl fluroide (Beyotime Institute of Biotechnology) were used for isolating the total protein. The lysates were cleared by centrifugation at 12,000 × g for 10 min at 4°C. Equal amounts of protein from each sample (30 μg) were separated by 8–12% SDS-PAGE and then transferred to polyvinylidine fluoride membranes (EMD Millipore, Billerica, MA, USA). The membranes were blocked with Tris-buffered saline (TBS; 10 mM Tris, pH 7.5, 100 mM NaCl), containing 5% non-fat dry milk, followed by an overnight incubation at 4°C with the primary antibodies. The primary antibodies used were against: SIRT1 (1:400 dilution; Santa Cruz Biotechnology, Inc., Santa Cruz, CA, USA), ORP150 (1:1,000 dilution; Epitomics, Burlingame, CA, USA), caspase 12 (1:1,000 dilution; Abcam, Cambridge, MA, USA), CHOP (1:500 dilution; Cell Signaling Technology, Inc., Danvers, MA, USA), caspase 4 (1:500 dilution; Protein Technologies, Manchester, UK), active-caspase 3 (1:1,000 dilution; Abcam). Rabbit anti-GAPDH was used as a control (1:1,000 dilution; Good HERE Biology, Hangzhou, China). The following day, the membranes were incubated with horseradish peroxidase-conjugated secondary antibodies (1:1,000 dilution; Protein Technologies) in TBS with Tween^®^. The blots were visualized using an enhanced chemiluminescence detection system (Advansta, Menlo Park, CA, USA). Each assay was performed in triplicate.

### Lentivirus conduction and stable transfection of human bronchial epithelial cells

Based on findings from the present study, an ORP150-specific small hairpin (sh)RNA was selected from a total of three candidate small interfering (si)RNAs targeting ORP150 mRNA. The selected shRNA had the highest ORP150 gene inhibitory effect, as determined by western blot analysis and qPCR. The sequence of the ORP150-specific siRNA, and the negative control siRNA were as follows: ORP150 siRNA, CATGGAAATTGTCTTGAAT; negative control siRNA, TTCTCCGAACGTGTCACGT. The oligonucleotides were designed according to the structure of the siRNA, the sequences were as follows: ORP150 sense, 5′-CCCATGGAAATTGTCTTGAAT-3′ and antisense, 5′-ATTCAAGACAATTTCCATGGG-3′; negative control sense, 5′-TTCTCCGAACGTGTCACGT-3′ and antisense, 5′-ACGTGACACGTTCGGAGAA-3′. The oligonucleotides containing both the ORP150 and control siRNA sequences were cloned into the GV118-green fluorescent protein (GFP) and GV112 vectors, to produce recombinant lentiviral vectors. The recombinant vectors were then cotransduced into human embryonic kidney 293T cells (Shanghai Institutes for Biological Sciences, Shanghai, China) using Lipofectamine^®^ 2000 (Invitrogen Life Technologies, Carlsbad, CA, USA). The human embryonic kidney 293T cells were maintained in Dulbecco’s modified Eagle’s medium supplemented with 10% heated FBS, 2 mM glutamine (Gibco-BRL), 100 U/ml penicillin and 100 μg/ml streptomycin (SV30010; Hyclone Laboratories, Inc.) at 37°C in an atmosphere of 5% CO_2_. Cells at 85–90% confluence were passaged by trypsinization. The supernatants containing the lentiviruses that expressed either the ORP150-specific shRNA or the control shRNA were harvested 48 h after transfection. The lentivirus was then purified by ultracentrifugation; the final titer of recombinant virus was 108 TU/ml.

Cultured normal HBEpC were infected with the ORP150 shRNA lentivirus, with a multiplicity of infection value of 100. After co-incubation with ORP150 RNAi for 8 hours, the cell culture media was refreshed. The transfection efficiency was confirmed after three days, based on fluorescence expression levels.

### Statistical analyses

The results are presented as the means ± standard deviation. SPSS version .17.0 (SPSS, Inc, Chicago, IL, USA) was used for statistical analyses. A one-way analysis of variance was used to determine the statistical significance of the measurement data, and a least significantly different t-test was used for comparison between two groups. A P≤0.05 was considered to indicate a statistically significant difference.

## Results

### Establishment of a CSE-induced apoptosis cell model in HBEpC

**Figure 1:**
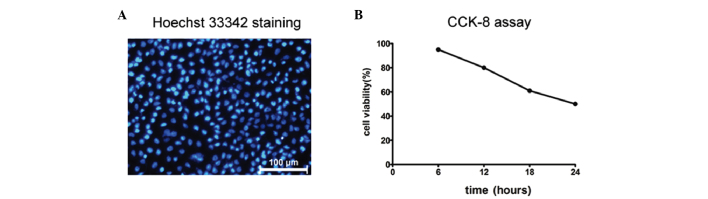
Effects of cigarette smoke extract (CSE) on cell viability and apoptosis in human bronchial epithelial cells (HBEpC). (A) HBEpC undergoing 2% CSE exposure for 24 h showed typical characteristics of apoptosis, as detected by Hoechst 33342 staining. Apoptotic cells showed strong fluorescent signals (bright turquoise colour), outlining the chromatin condensed nuclei, whereas normal cells appeared weakly stained (magnification, ×400). (B) Cell counting kit-8 (CCK-8) assay showed that following 2% CSE exposure for 24 h, the cell viability of HBEpC declined. H, hours.

Consistent with previous reports (21,23), HBEpC underwent apoptosis after being exposed to 2% CSE (volume of CSE/volume of medium) for 6–24 h, as detected by Hoechst 33342 staining (Fig. 1A). A CCK-8 assay (Fig. 1B) confirmed that at the end of the 24 h 2% CSE exposure, the survival rate of HBEpC was ≥50%, which was suitable for showing any significant cell protective effects using this cell model.

### Protective effects of RES against CSE-induced apoptosis and the expression of SIRT1 and ORP150 genes

**Figure 2:**
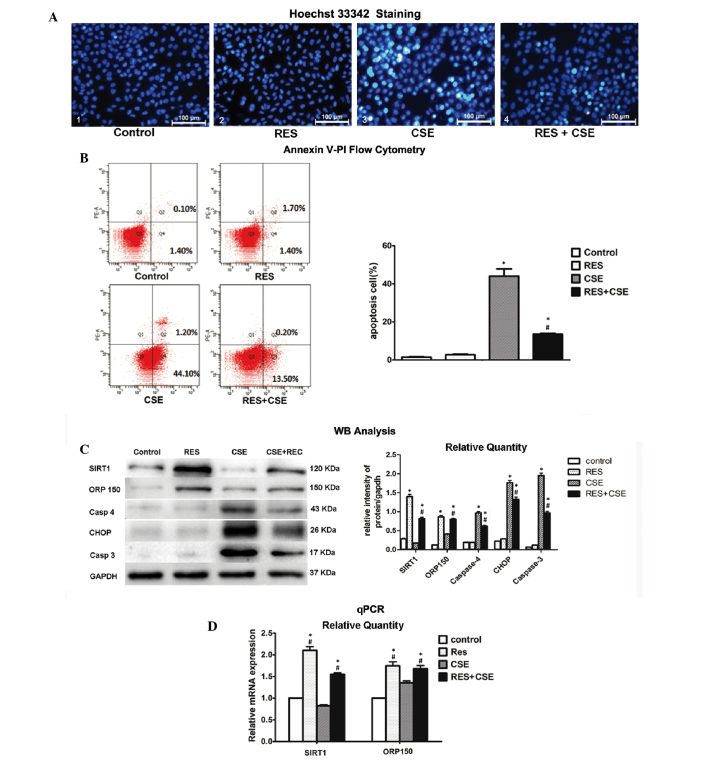
The protective effects of resveratrol (RES) against cigarette smoke extract (CSE)-induced apoptosis and the activity of Sirtuin 1 (SIRT1) and oxygen-regulated protein 150 (ORP150) genes (A) Hoechst staining of human bronchial epithelial cells (HBEpC). HBEpC pre-treated with RES, exhibited fewer apoptotic cells, as compared with the non-RES treated group, following a 24 h exposure to 2% CSE. Apoptotic cells are strongly stained bright turquoise (magnification, ×400). (B) Annexin V-PI flow cytometry confirmed that 24 h 2% CSE exposure caused severe cell damage, with ~45.3% apoptotic cells. With RES pre-treatment, the apoptotic cell count significantly decreased, indicating that RES may attenuate CSE-induced apoptosis in HBEpC. (C) Western blot analysis showed that RES induced the relative protein expression levels of Sirtuin 1 (SIRT1) and oxygen-regulated protein 150 (ORP150) in the RES and RES + CSE groups. CSE induced an upregulation in the protein expression levels of apoptosis markers caspase 4, CCAAT-enhancer binding protein homologous protein (CHOP) and caspase 3, and RES attenuated the overexpression of those markers in the RES+CSE group, as compared with the CSE group. (D) Quantitative polymerase chain reaction (qPCR) demonstrated a simultaneous overexpression of SIRT1 and ORP150 at the mRNA level following RES pre-treatment of HBEpC, indicating that RES may activate both SIRT1 and ORP150. ^*^P<0.05 vs. control groups; ^#^P<0.05 vs. CSE group. The data are presented as the means ± standard deviation. kDa, kilodaltons.

In the present study, HBEpC were divided into four groups: 1) Control, HBEpC at 80% confluence cultured without CSE or RES; 2) RES, 20 μmol/l RES added to the cell culture media; 3) CSE, HBEpC underwent 2% CSE exposure, for 24 h; 4) RES+CSE, HBEpC were pre-cultured with 20 μmol/l RES for 2 h, followed by a 24 h exposure to 2% CSE. A 24 h 2% CSE exposure resulted in a typical apoptotic change in the cells, as compared with the cells from the other groups (Fig. 2A). RES pre-culture markedly alleviated the severity of apoptosis, which was confirmed by Hoechst 33342 staining and Annexin V-PI flow cytometry (Fig. 2B). Following Hoechst 33342 staining, fewer positive apoptotic cells were observed in the RES pre-treated cells, and Annexin V-PI showed a significant decrease in the apoptotic cell rate of the RES pre-treated cells, as compared with the cells without RES treatment (13.7 vs. 45.3%; P<0.05).

At the molecular level, the activation of both SIRT1 and ORP150 by RES, and the consequently downregulated expression levels of the apoptotic markers caspase 3, caspase 4 and CHOP, were observed in Fig. 2C and D. During apoptosis of the cells of the CSE group, the relative protein expression levels of the apoptotic markers were overexpressed, as detected by western blotting analysis. However, when the cells were pre-cultured with RES, there was a significant upregulation in the relative mRNA and protein expression levels of SIRT1 and ORP150 (Fig. 2C and D), accompanied by a significant downregulation of the expression levels of the apoptotic markers. These results indicate that RES had protective effects against CSE-induced apoptosis in HBEpC.

### ORP150 gene knockdown attenuates the protective effects of RES against CSE-induced apoptosis in HBEpC

**Figure 3:**
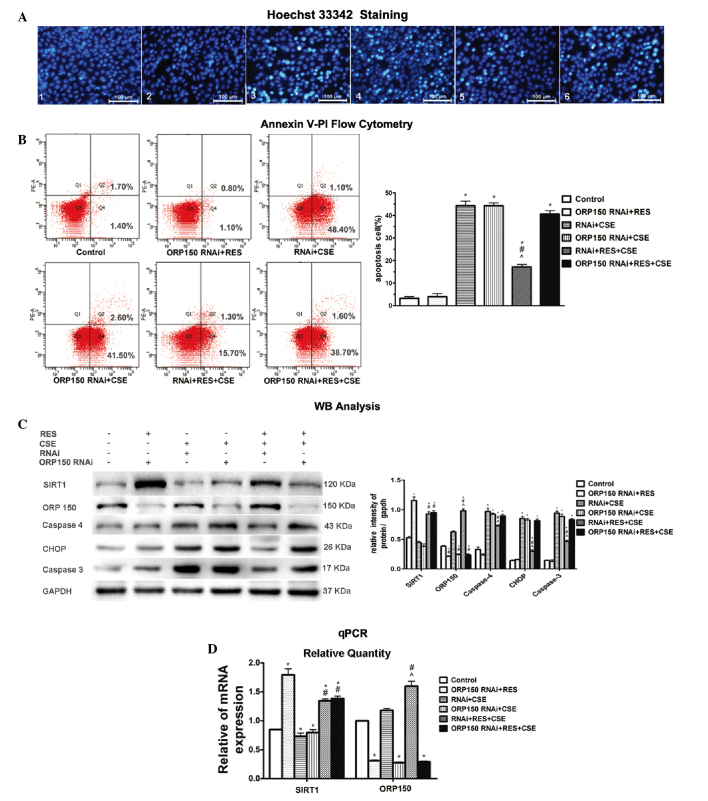
ORP150 gene knockdown attenuated the protective effects of resveratrol (RES) against cigarette smoke extract (CSE)-induced apoptosis in human bronchial epithelial cells (HBEpC) (A1–6) Hoechst 33342 staining of the experimental groups. (A3) The 24 h 2% CSE exposure lead to significant apoptosis (bright turquoise stained cells, whereas (A5) RES markedly prevented the cells from undergoing apoptosis. (A6) The protective effects of RES were attenuated by the knockdown of ORP150 by RNA interference (RNAi), as compared with (A5) the cells transfected with control RNAi (magnification, ×400). (B) The apoptotic cell count detected by flow cytometric analysis of Annexin V-PI clearly confirmed the findings obtained from Hoechst 33342 staining. The CSE-induced apoptosis was markedly attenuated by RES pre-treatment (RNAi + RES + CSE); however, this protective effect was abolished by ORP150 gene knockdown (ORP150 RNAi + RES + CSE). (C) Western blot analysis showed the relative protein expression levels of Sirtuin 1 (SIRT1), oxygen-regulated protein 150 (ORP150), and the apoptosis marker molecules in all of the groups. CSE exposure caused upregulation of the apoptosis marker molecules caspase 4, CCAAT-enhancer binding protein homologous protein (CHOP) and caspase 3. SIRT1 and ORP150 expression were increased following RES pre-treatment. The knockdown of ORP150 eliminated the protective effects of RES as shown in the ORP150 RNAi + RES + CSE group, as compared with the RNAi + RES + CSE group. (D) Quantitative polymerase chain reaction (qPCR) showed relative SIRT1 and ORP150 mRNA expression levels. ORP150 shRNA significantly decreased the expression of ORP150 mRNA in HBEpCs transfected with OPR150 RNAi, as shown in groups 2, 4 and 6, whereas control shRNA was not effective at all. SIRT1 gene expression was not affected by the knockdown of ORP150 gene.^*^P<0.05 vs control group. ^#^P<0.05 vs RNAi + CSE group. ^^^P<0.05 vs ORP150 RNAi + RES + CSE. The data are presented as the means ± standard deviation. kDa, kilodaltons.

To determine whether ORP150 was associated with the protective effects of SIRT1 against CSE-induced apoptosis in HBEpC, an ORP150 gene knockdown was performed (Fig. 3). HBEpC were divided into six groups: 1) Control, HBEpC at 80% confluence cultured without any treatment for 24 h; 2) ORP150 RNAi+RES, ORP150 shRNA-transduced HBEpC, pre-cultured with 20 μmol/l RES; 3) RNAi + CSE, negative control shRNA-transduced HBEpC exposed to 2% CSE for 24 h; 4) ORP150 RNAi + CSE, ORP150 shRNA-transduced HBEpC exposed to 2% CSE for 24 h; 5)RNAi + RES + CSE, negative control shRNA-transduced HBEpC, pre-cultured with 20 μmol/l RES for 2 h, followed by a 24 h exposure to 2% CSE; 6) ORP150 RNAi + RES + CSE, ORP150 shRNA-transduced HBEpC, pre-cultured with 20 μmol/l RES for 2 h, followed by a 24 h exposure to 2% CSE.

Western blot analysis demonstrated that following HBEpC exposure to CSE (groups 3, 4, 5 and 6), there was a marked upregulation of the apoptotic markers CHOP, caspase 4 and caspase 3. Both Hoechst 33342 staining and Annexin V-PI flow cytometry (Fig. 3A and B) demonstrated similar changes in the severity of the apoptosis among the differently treated cell groups. Following exposure to CSE, HBEpC in groups 3, 4 and 6 suffered obvious damage, with a significant portion of the cells positively stained by Hoechst 33342 staining (Fig. 3A), and a high apoptotic cell rate (Fig. 3B). The protective effects of RES were still present in the cells that were transfected with the negative control shRNA, undergoing CSE exposure (group 5). However, following transfection of HBEpC with the specific ORP150 shRNA (group 6), the protective effects of RES were markedly attenuated (Fig. 3C). The cells pre-incubated with control shRNA showed no effect on ORP150 expression, implying that a successful knockdown of the targeted ORP150 gene was constructed in HBEpC (Fig. 3D). As compared with group 6, the cells in group 5 showed marked durability against CSE-induced apoptosis when the cells were transfected with negative control siRNA, and downregulation of the apoptotic markers CHOP, caspase 4 and caspase 3 (Fig. 3C), thus implying that the protective effects of RES on the cells may be achieved by expression of ORP150. The apoptotic cell rate in group 5 was 17.0%, as compared with 49.5, 44.1 and 40.3% in groups 3, 4 and 6. However, the protective effects of RES were markedly reduced following the shRNA knockdown of ORP150, as shown in group 6. An increased number of apoptotic cells were detected, together with a much higher apoptotic rate and a marked upregulation of the apoptotic marker molecules (Fig. 3C), as compared with group 5.

These results indicate that ORP150 is necessary for RES to exert its anti-apoptotic effects in the CSE apoptosis cell model. However, the mechanisms by which SIRT1 interacts with ORP150, remains unclear.

## Discussion

CS typically causes ER-stress, which may eventually lead to apoptosis. Our previous study demonstrated that cigarette smoke may induce GRP78 expression in A549 cells, and the upregulated GRP78 expression in the cells may have an anti-apoptotic effect (24). Human bronchial epithelial cells are the first line of defense against external pathogens in the respiratory tract. Excessive apoptosis of the epithelial cells of the airways and defective repair processes are hallmarks of COPD (25). In the CS-induced apoptotic HBEpC model used in the present study, the apoptosis of HBEpC was confirmed to be due to ER-stress, as shown by the upregulation of the apoptotic markers caspase 4 and CHOP, which was consistent with the findings of Tagawa *et al* (26). SIRT1 exerts anti-inflammatory and anti-aging effects in the pathogenesis of COPD. RES, which is a natural activator of SIRT1, is currently under investigation as an alternative in COPD therapy, which may focus on the anti-oxidative and anti-ageing effects of RES. However, whether RES regulates endoplasmic reticulum stress-induced apoptosis, particularly in the development of COPD remains unclear. In 2012, Yao *et al* (27) revealed that the level of SIRT1 is significantly decreased in the lungs of COPD patients, as well as the lungs of rodents exposed to cigarette smoke. In agreement with these findings, in the present study, using the HBEpC apoptosis model, the levels of SIRT1 were decreased in the HBEpC following exposure to CSE, as compared with the normal HBEpC (control group). When HBEpC were pre-cultured with RES, there was an upregulation of the relative mRNA and protein expression levels of both SIRT1 and ORP150. The parameters that measured the occurrence and severity of apoptosis also showed that following RES pre-culture, the cells had a lower apoptotic cell rate and fewer apoptotic cells were detected. These findings were accompanied by a down-regulation of the relative protein expression levels of apoptosis marker molecules caspase 3, caspase 4, and CHOP.

The anti-apoptotic effects of RES observed in the present study are of particular relevance, since ER-stress and ER-stress-induced apoptosis are the primary triggers for the development of COPD (15,28,29). Treatments that alleviate the severity of ER-stress or ER-stress induced apoptosis would be highly useful in the development of a new therapy for COPD.

To elucidate the mechanism behind the anti-apoptotic effects of RES in the CSE-HBEpC model is beyond the realm of the present study. However, the findings of the present study clearly indicate that RES is capable of protecting HBEpC against CSE-induced apoptosis through a pathway involving both SIRT1 and ORP150. RES was shown to induce upregulation of both SIRT1 and ORP150 mRNA and protein expression levels in HBEpC, which was strongly associated with the decrease in apoptosis. Furthermore, the ORP150 gene knockdown abolished the protective effects of RES on CSE-induced apoptosis of HBEpC.

In conclusion, the results of the present study demonstrate that RES has a protective effect against CSE-induced apoptosis in HBEpC. This anti-apoptotic effect may be exerted through the activation of a pathway involving SIRT1 and ORP150. Considering that apoptosis and senescence have a crucial role in the pathogenesis of COPD, the therapeutic effects of RES shows significant potential for the prevention and treatment of COPD.
